# Reduction of Neuropsychiatric Symptoms and Managing Safety Outcomes by Pimavanserin Versus Other Antipsychotics: Systematic Review and Meta Analysis.

**DOI:** 10.1192/bjo.2025.10197

**Published:** 2025-06-20

**Authors:** Sneh Babhulkar, Sathyan Soundara Rajan, Gaurav Uppal, Maryum Maryum, Ugo Okafor

**Affiliations:** ^1^Gartnavel Royal Hospital, Glasgow, United Kingdom; ^2^BCUHB, Wrexham, United Kingdom; ^3^Satyam Hospital, Ludhiana, India; ^4^Tallaght University Hospital, Dublin, Ireland; ^5^Mater Misericordiae Hospital, Dublin, Ireland

## Abstract

**Aims:** This meta-analysis aims to review safety and effectiveness of pimavanserin compared with other antipsychotics in managing psychotic symptoms in Alzheimer’s disease and Parkinson’s disease dementia.

**Methods:** We conducted a comprehensive literature search of controlled trials evaluating efficacy of pimavanserin versus placebo and other antipsychotics. A thorough search was made using specific terms in Pubmed, Web of Science, EMBASE, SIGLE and CINAHL. Of 423 studies only 2 studies met our requirements once detailed ROB 2 analysis was performed. The primary dependent measure was NPI and the CGI-I as the secondary measure;safety data being the other dependent measure.

**Results:** With active treatment by pimavanserin, there was a mean reduction of 4.5 points on NPI score, the SMD was −1.07 compared with placebo. It was more effective than other antipsychotics and it came with more acceptable side effects. Side effects included extrapyramidal symptoms, however this was significantly lower in the pimavanserin group (7% versus 15% with olanzapine) and minimal metabolic side effects.

**Conclusion:** Pimavanserin stands as a relatively new treatment approach for management of neuropsychiatric symptoms in dementia that has similar effectiveness when compared with other antipsychotics, yet exhibiting fewer side effects. Its mechanism of action as a selective serotonin inverse agonist may offer some advantage in controlling and managing psychotic symptoms without worsening of motor functions in patients with Parkinson’s disease dementia.

